# Dynamic characteristics of a COVID-19 outbreak in Nanjing, Jiangsu province, China

**DOI:** 10.3389/fpubh.2022.933075

**Published:** 2022-11-22

**Authors:** Junjun Wang, Tao Ma, Songning Ding, Ke Xu, Min Zhang, Zhong Zhang, Qigang Dai, Shilong Tao, Hengxue Wang, Xiaoqing Cheng, Min He, Xuefei Du, Zhi Feng, Huafeng Yang, Rong Wang, Chaoyong Xie, Yuanyuan Xu, Li Liu, Xupeng Chen, Chen Li, Wen Wu, Sheng Ye, Sheng Yang, Huafeng Fan, Nan Zhou, Jie Ding

**Affiliations:** ^1^Nanjing Municipal Center for Disease Control and Prevention, Nanjing, China; ^2^Chinese Field Epidemiology Training Program, Chinese Center for Disease Control and Prevention, Beijing, China; ^3^Department of Acute Infectious Diseases Control and Prevention, Nanjing Municipal Center for Disease Control and Prevention, Nanjing, China; ^4^Department of Acute Infectious Diseases Control and Prevention, Jiangsu Provincial Center for Disease Control and Prevention, Nanjing, China; ^5^Jiangning District Center for Disease Control and Prevention, Nanjing, China

**Keywords:** coronavirus disease 2019 (COVID-19), SARS-CoV-2, B.1.617.2 (Delta) variant, transmissibility, serial interval, reproduction number

## Abstract

**Objectives:**

Severe acute respiratory syndrome coronavirus 2 (SARS-CoV-2) lineage B.1.617.2 (also named the Delta variant) was declared as a variant of concern by the World Health Organization (WHO). This study aimed to describe the outbreak that occurred in Nanjing city triggered by the Delta variant through the epidemiological parameters and to understand the evolving epidemiology of the Delta variant.

**Methods:**

We collected the data of all COVID-19 cases during the outbreak from 20 July 2021 to 24 August 2021 and estimated the distribution of serial interval, basic and time-dependent reproduction numbers (R_0_ and R_t_), and household secondary attack rate (SAR). We also analyzed the cycle threshold (Ct) values of infections.

**Results:**

A total of 235 cases have been confirmed. The mean value of serial interval was estimated to be 4.79 days with the Weibull distribution. The R_0_ was 3.73 [95% confidence interval (CI), 2.66–5.15] as estimated by the exponential growth (EG) method. The R_t_ decreased from 4.36 on 20 July 2021 to below 1 on 1 August 2021 as estimated by the Bayesian approach. We estimated the household SAR as 27.35% (95% CI, 22.04–33.39%), and the median Ct value of open reading frame 1ab (ORF1ab) genes and nucleocapsid protein (N) genes as 25.25 [interquartile range (IQR), 20.53–29.50] and 23.85 (IQR, 18.70–28.70), respectively.

**Conclusions:**

The Delta variant is more aggressive and transmissible than the original virus types, so continuous non-pharmaceutical interventions are still needed.

## Introduction

Severe acute respiratory syndrome coronavirus 2 (SARS-CoV-2), first identified in December 2019, is constantly changing throughout the global pandemic of coronavirus disease 2019 (COVID-19). The outbreaks caused by emerging variants have been documented globally, and scientific evidence is growing that certain variants are associated with higher transmissibility, disease severity, and immune escape abilities (1, 2). The emerging variant is classified as a variant of concern (VOC) or a variant of interest (VOI) based on its characteristics, which may exert some impact on related countermeasures (1). The Delta (B.1.617.2) variant of SARS-CoV-2 was designated as a VOC on 11 May 2021, caused the resurgence of COVID-19 cases in India, and gradually became the epidemic strain in India, Singapore, the UK, and Australia (3, 4). As of November 26, 2021, five VOC variants have been identified, including Alpha (B.1.1.7), Beta (B.1.351), Gamma (P.1), Delta (B.1.617.2), and Omicron (B.1.1.529) (1).

The Delta variant possesses more powerful transmissibility and/or evolutionary advantages to escape host immunity compared with the non-VOC/VOI variants and other previously reported VOC variants such as Alpha (B.1.1.7) (3, 5, 6). In May 2021, Guangdong province reported the first local outbreak with the Delta variant as the etiologic pathogen in China (7). On 20 July 2021, nine positive results in mixed screening samples were detected during routine nucleic acid assays for airport staff at Nanjing Lukou International Airport. Locally transmitted cases linking to the airport were reported in the following days and the outbreak was identified to have been caused by the Delta variant by the detection of clustered Delta genome sequences.

This study aimed to describe the outbreak triggered by the Delta variant through the transmission parameters and to provide more information for estimating the impact of control measures on outbreaks of the Delta variant. The emergence of novel variants of concern [e.g., Alpha (B.1.1.7), Beta (B.1.351), and Delta (B.1.617.2) variants] can be expected to change the characteristics of the transmission. The description of this Delta variant outbreak helps to provide a reference for exploring the transmissibility of the new emerging variant [Omicron (B.1.1.529) variant] (8) or future unknown variants of SARS-CoV-2 and gain an insight into the countermeasure of controlling the ongoing COVID-19 pandemic.

## Methods

### Study population

We retrospectively collected information on all laboratory-confirmed cases of the Delta variant infection from the outbreak in Nanjing city from July to August 2021. The COVID-19 case was diagnosed based on the “*China's guidelines for diagnosis and treatment of COVID-19* (Trial Version 8)” released by the National Health Commission of the People's Republic of China, and the degree of clinical severity of COVID-19 was classified as mild, moderate, severe, or critical.

### Specimen collection and testing

All cases were confirmed with the positive detection of SARS-CoV-2 nucleic acid. During the isolation or quarantine period, cases and close contacts received regular specimen collection, testing, and daily health surveillance. Reverse transcription-polymerase chain reaction (RT-PCR) was applied for SARS-CoV-2 nucleic acid detection in respiratory specimens by eligible healthcare facilities, local Centers for Disease Control and Prevention (local CDCs), or third-party medical inspection institutions. Laboratory confirmation of the diagnosis was performed at the Nanjing Municipal Center for Disease Control and Prevention (NJCDC).

### Data collection and statistical analysis

According to the “*Protocol for Prevention and Control of COVID-19* (Edition 8)” released by the National Health Commission of the People's Republic of China, epidemiological surveys were implemented for all the cases. Epidemiological investigation reports on all cases were developed and updated in a timely manner. The timeline of key events was extracted, including the date of exposure, symptom onset, sample collection, being placed under quarantine, or being admitted to the designated hospital, and so on. The clinical severity information was collected from the online direct reporting system [National Notifiable Disease Surveillance System (NNDSS)] on 8 September 2021.

The epidemic curve was constructed using the onset date of symptomatic cases. We explored the chains of transmission based on the time of exposure, the sources of infection, the date of symptom onset, laboratory testing results, etc. Based on the defined chains of transmission, we identified infector–infectee pairs, and the infectee (known as the consecutive case) has a defined exposure history, i.e., exposure to a single infector (known as the primary case) without other infection sources. We estimated the serial interval by identifying the duration (days in this study) of symptom onset between the primary case and the consecutive case in all infector–infectee pairs. An index case in a household was defined as the confirmed case with the earliest date of symptom onset in the household (9). A household close contact is defined as a person living in the same household with the index case without effective protection within 2 days before the symptom onset of a symptomatic index case or 2 days before the first positive sample was collected from an asymptomatic index case. We also measured household transmission through the household secondary attack rate (SAR). Household SAR was determined by the number of new cases (secondary cases) identified among household close contacts exposed to household index cases.

The basic reproduction number (R_0_) measures the average number of secondary cases in a susceptible population infected by a typical primary case during the infection period (10). Based on the estimation of the serial interval distribution and the epidemic curve of new daily cases, R_0_ was estimated using the maximum likelihood (ML) and the exponential growth (EG) methods, respectively. We conducted a sensitivity analysis with deviance R-squared statistic to select the optimal period of the epidemic curve that best fitted exponential growth (11). Compared with R_0_, which gives an indication of the spreading potential of the virus in a susceptible population, the time-dependent reproduction number (R_t_) reflects the real-time transmissibility of infection on day t, reflecting the effectiveness of multiple control measures in reducing transmission (11–13). We used the Bayesian approach to estimate the R_t_ value over sliding weekly windows (12).

A continuous variable was represented as the median and interquartile range (IQR). A categorical variable was represented as the count and percentage. Standard non-parametric tests (the Kruskal–Wallis test and Mann–Whitney *U* test) were used to compare the characteristics between demographic groups. The serial interval, R_0_ value, and R_t_ value were estimated by using the R software version 4.0.5 [with the package “R0” version 1.2–6 (11) and package “EpiEstim” version 2.2–4 (12)]. Other analyses were performed with SPSS Statistics 21.0 software. The 95% confidence interval (CI) was used to assess statistical significance.

## Results

**Figure 1:**
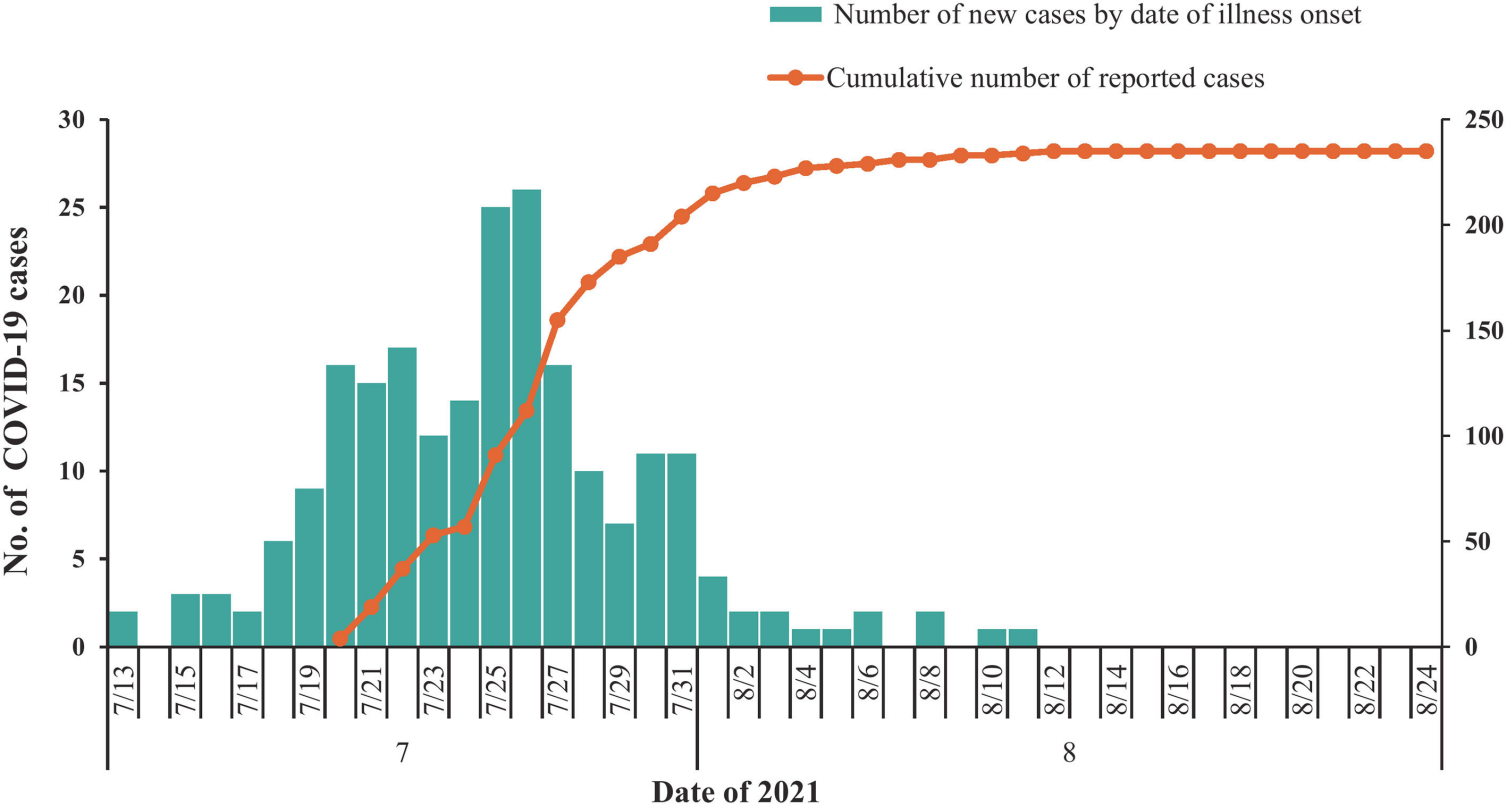
Temporal distribution of confirmed coronavirus disease 2019 (COVID-19) cases (*N* = 235) in Nanjing, July to August 2021. Date of illness onset: date of illness onset according to the epidemiological investigation reports.

As of 24 August 2021, a total of 235 COVID-19-infected cases were identified in the outbreak in Nanjing (Figure 1). Ninety-four (40.0%) of these affected patients were men. The median age of all patients was 44.0 years (IQR: 34.0–53.0), with 28 (11.9%) of them younger than 20 years and 33 (14.0%) of them older than 59 years. The number of moderate, mild, severe, and critical cases was 166 (70.64%), 59 (25.11%), 9 (3.83%), and 1 (0.42%), respectively.

**Figure 2:**
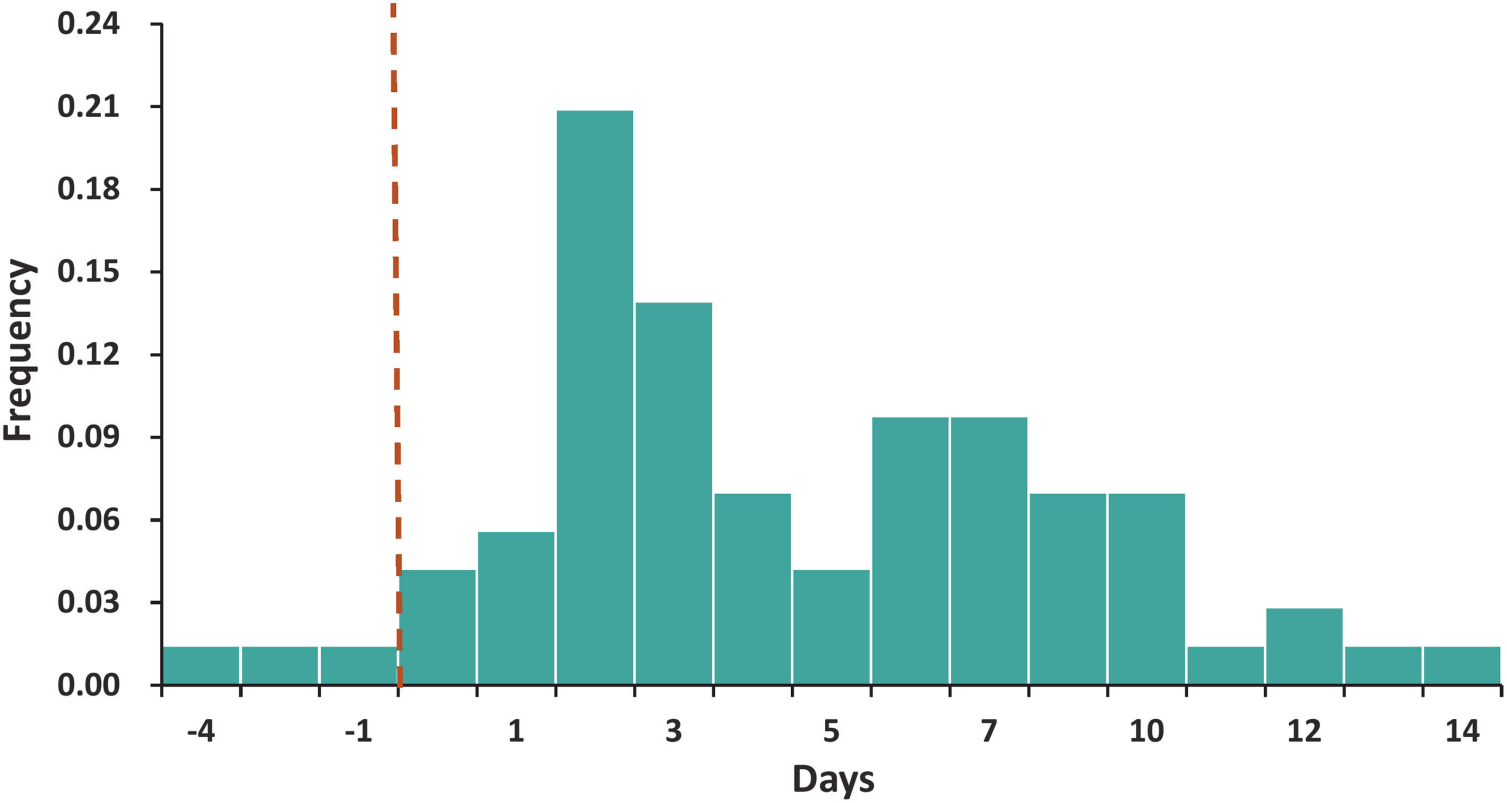
The distribution of the serial interval. The best-fitting distribution of the serial interval by Weibull distributions based on 72 transmission pairs. Negative serial intervals, which signify pre-symptomatic transmission, were also included in the analysis.

Based on epidemiological evidence, defined transmission chains were identified in 72 infector–infectee pairs clearly, with the included recipients having no history of contact with other confirmed cases. The mean serial interval was 4.65 days (95% CI, 3.79–5.52). Under the fitted Weibull distribution, the serial interval distribution adjusted to our observed data was determined as a mean of 4.79 days and a standard deviation (SD) of 3.47 days (Figure 2). There was no significant difference (*P* > 0.05) in the serial interval between the age groups of the infectors. Among the infectors aged 20–59 years, 67 infector–infectee pairs were identified and the mean serial interval was 4.85 (SD: 3.60) days with a fitted Weibull distribution. Among the infectors aged 60 years or older, 5 infector–infectee pairs were identified and the mean serial interval was 3.97 (SD: 1.35) days with a fitted lognormal distribution. The difference in serial interval between male and female infector cases also showed no statistically significant value (*P* > 0.05). Among male infectors, 12 infector–infectee pairs were identified and the mean serial interval was 3.93 (SD: 5.49) days with a fitted lognormal distribution. Among female infectors, 60 infector–infectee pairs were identified and the mean serial interval was 5.02 (SD: 3.44) days with a fitted Weibull distribution.

**Figure 3:**
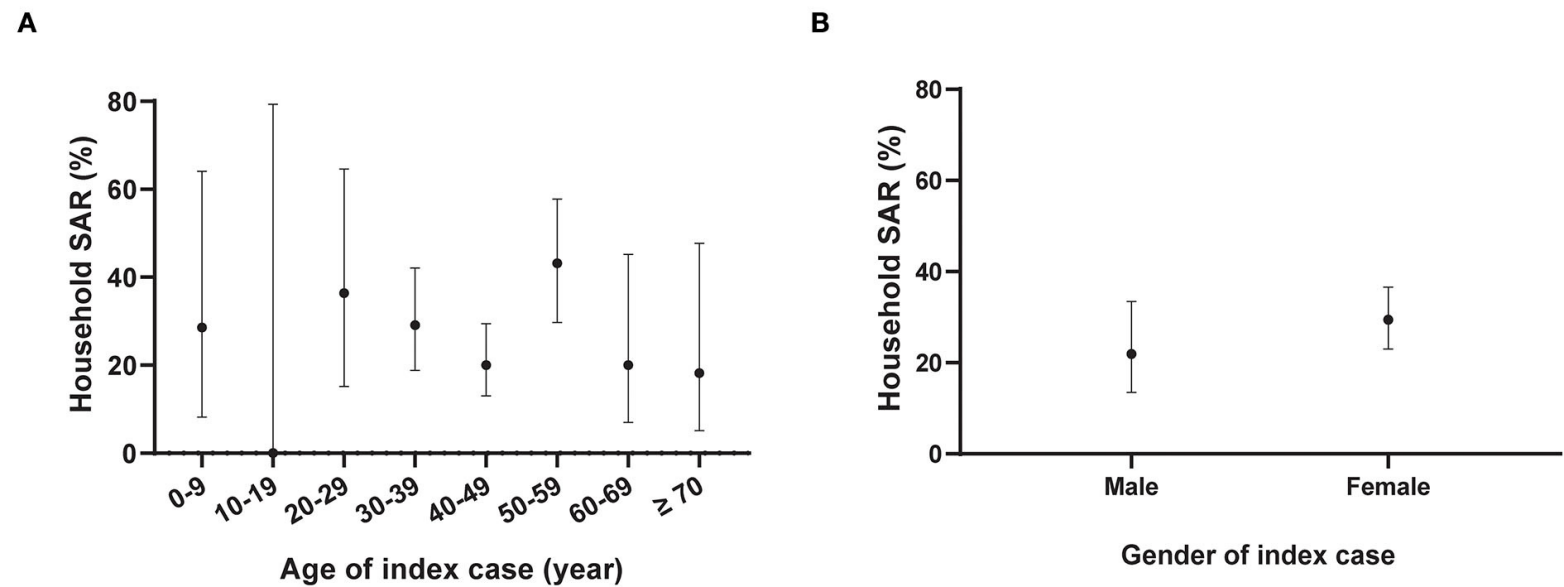
Household secondary attack rates of Severe acute respiratory syndrome coronavirus 2 (SARS-CoV-2) by gender and age distribution of the index cases. *N* = 234 household contacts; SAR: Secondary attack rate. The black dot represents the estimate of household SAR among household contacts stratified by age **(A)** or gender **(B)** of the index cases; the vertical black line represents a 95% confidence interval (CI). The age group of 10–19 years included one household contact without COVID-19 infection.

We identified 234 household close contacts and 64 secondary cases. The household SAR was 27.35% (95% CI, 22.04–33.39%). The household SARs among household contacts exposed to index cases aged 0–9 years, 10–19 years, 20–29 years, 30–39 years, 40–49 years, 50–59 years, 60–69 years, and older than 69 years were 28.6% (2/7), 0.0% (0/1), 36.4% (4/11), 29.1% (16/55), 20.0% (18/90), 43.2% (19/44), 20.0% (3/15), and 18.2% (2/11), respectively. There was no significant association between the age distributions of the index cases with the household SARs (*P* > 0.05). The household SARs among household contacts exposed to male index cases and female index cases were 21.9% (14/64) and 29.4% (50/170), respectively. There was also no significant association determined between the household SAR and gender distribution of index cases (*P* > 0.05) (Figure 3).

**Figure 4:**
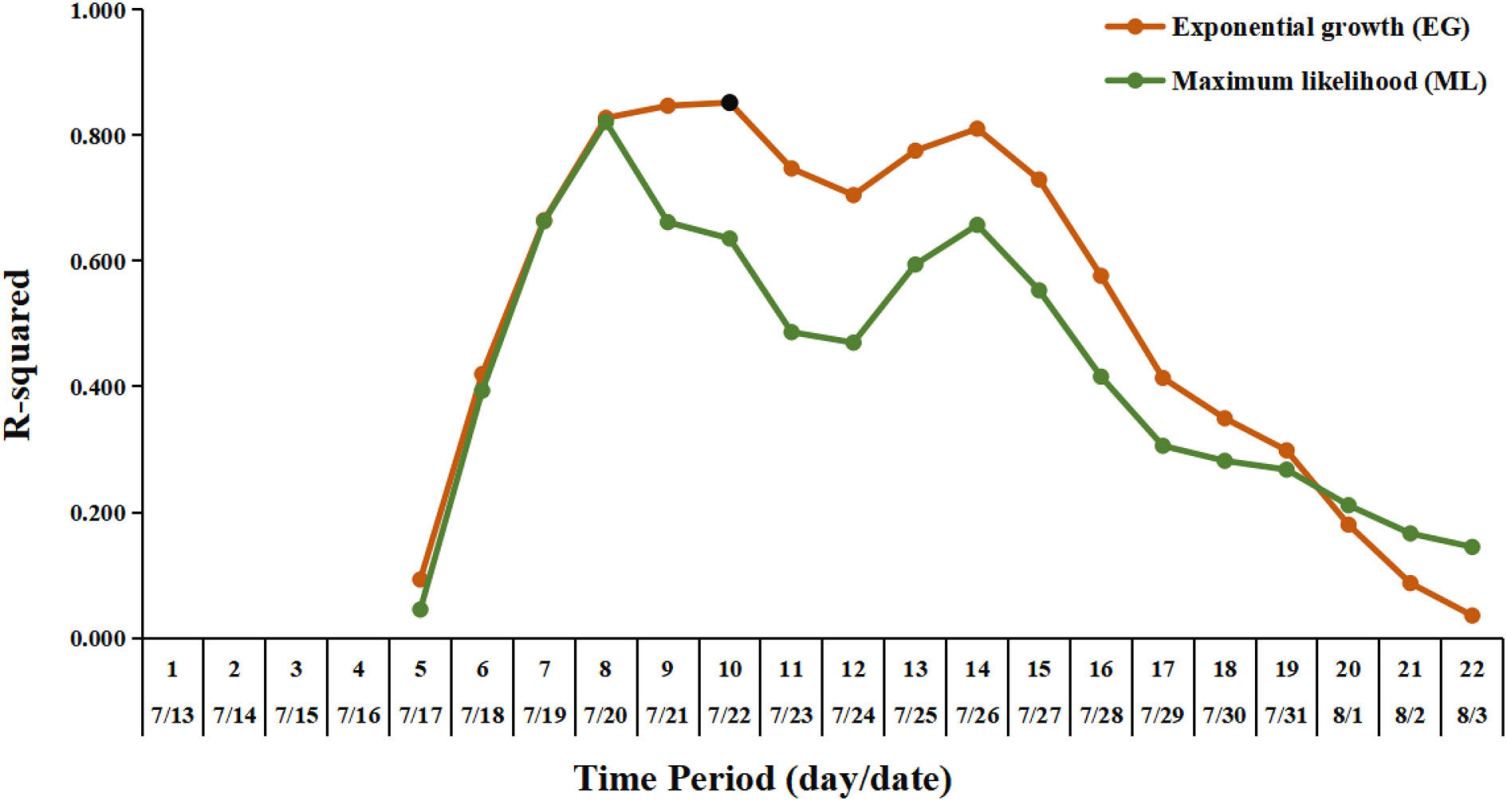
Exponential growth fitting results using deviance R-squared statistic for sensitivity analysis. The vertical axis shows the deviance R-squared values for the estimation of the R_0_. The horizontal axis shows the length of time periods, from the earliest date of symptom onset in this outbreak (July 13, 2021) to the possible selected date. The orange line and the dark green line indicate the fitting results for the estimation of the R_0_ by the exponential growth (EG) and maximum likelihood (ML) method, respectively. The black dot represents the value corresponding to the optimal time period with the length of time periods being 10 (from July 13 to July 22), for the estimation of the R_0_ by the EG method.

**Figure 5:**
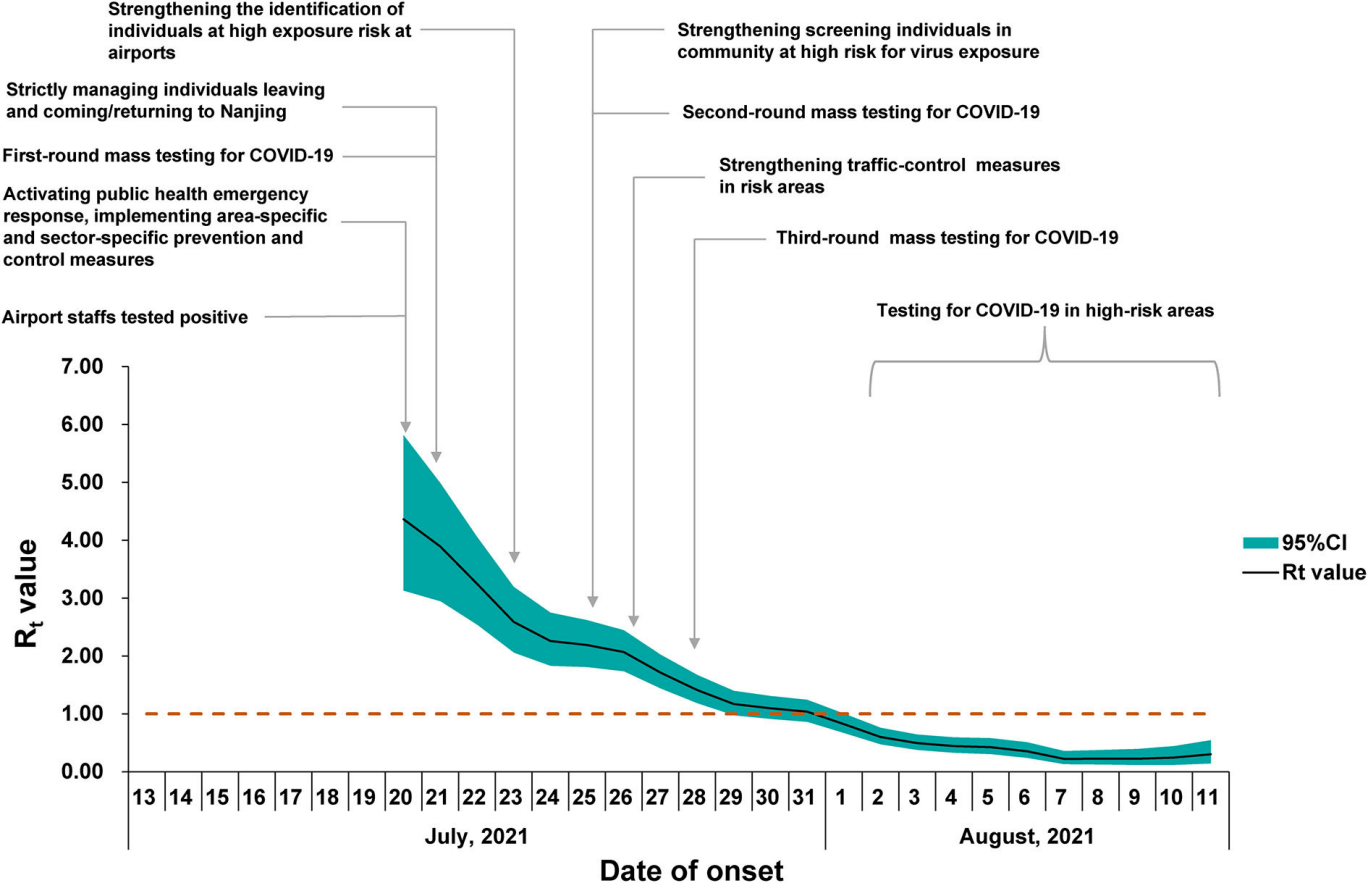
Time-dependent reproduction number (R_t_) for confirmed coronavirus disease (2019) (COVID-19) cases in Nanjing. The black line represents the mean estimate of R_t_ and the dark green area represents the 95% confidence intervals (CIs) of the estimates of R_t_ at time t. The horizontal dashed line indicates the threshold value R = 1. The timeline of the implemented non-pharmaceutical interventions (NPIs) is represented as gray vertical lines.

For the estimation of the R_0_, the sensitivity analysis showed that the EG method was fitted better than the ML method. The optimal period of the epidemic curve, best fitting the exponential growth with the EG method, was over the time window ranging from 13 July to 22 July (Figure 4). Using the EG method, the estimate of R_0_ was determined as 3.73 (95% CI, 2.66–5.15). The R_t_ value reached the peak value of 4.36 on 20 July 2021 and rapidly declined to lower than 1 on 1 August 2021, and then, it was maintained below 1 until 11 August 2021 (onset date of the last case) (Figure 5).

**Figure 6:**
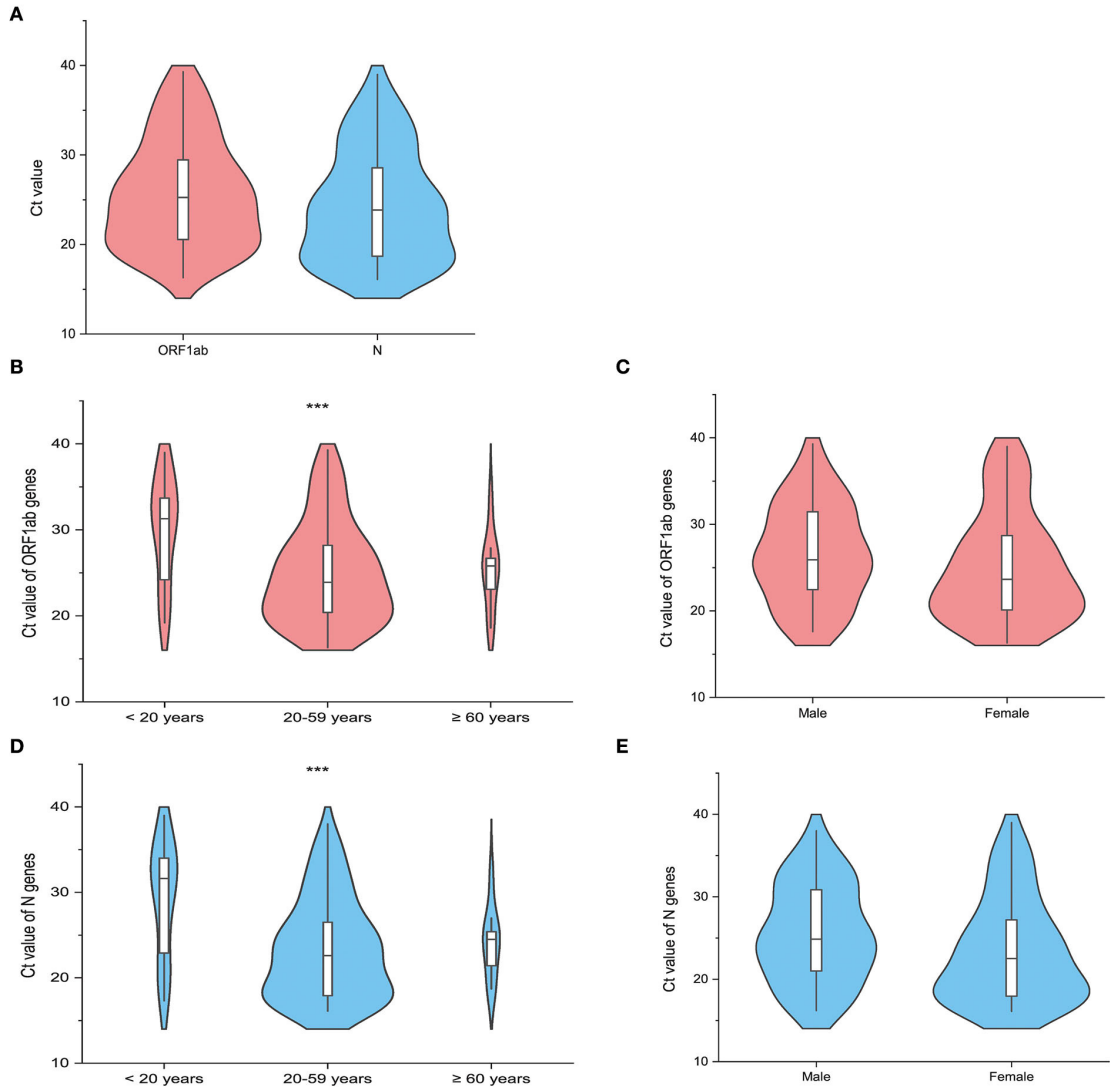
The distribution of the cycle threshold (Ct) values of the first reverse transcription-polymerase chain reaction (RT-PCR) positive results in the quarantined subjects (*N* = 92). The violin plot shows the distribution of the Ct values of the open reading frame 1ab (ORF1ab) gene (Orange red) and the Ct values of the nucleocapsid protein (N) gene (Light blue) among the included quarantined subjects (*N* = 92). The white bar in the center represents the interquartile range (IQR), and the black horizontal line in the white bar represents the median value. Whiskers below and above the white bar indicate the smallest and largest values no further than 1.5 times IQR from the 25th percentile or 75th percentile, respectively. **(A)** Shows the distribution of Ct values of target genes among all the included cases. The other violin plots show the difference in the Ct values of the target genes (ORF1ab gene and N gene) across different age groups **(B,D)**, including <20 years, 20–59 years, and ≥60 years, and gender groups **(C,E)**. *** represents the *P-*value with the non-parametric test to be lower than 0.05.

The RT-PCR was performed to detect the presence of COVID-19 ribonucleic acid (RNA), which provided Ct values of the specimens. Following the entry into host cells, a virus makes copies in the infection sites and sheds viral particles variably, which is associated with clinical status. Specimens were collected from an individual under quarantine who was required to receive daily RT-PCR tests for SARS-CoV-2. Only the Ct values of the first positive PCR results were analyzed in our study to ensure the comparability of Ct values at the earliest phase of infection. In addition, the time interval between the dates of specimen collection with the first positive PCR result and the dates of admission to the quarantine sites is at least 1 day to exclude PCR-positive results obtained before the time of quarantine. The Ct values of the two target genes, including the open reading frame 1ab (ORF1ab) gene and the nucleocapsid protein (N) gene, were tested at the same laboratory (NJCDC), using the same protocol including real-time fluorescent quantitative RT-PCR and the same commercial brand of COVID-19 coronavirus real-time PCR Kit (Jiangsu Bioperfectus Technologies Co., Ltd.). The specimens collected from 92 confirmed cases at the designated quarantine sites were subjected to the estimation of Ct values. The median Ct value of ORF1ab genes was 25.25 (IQR, 20.53–29.50) (Figure 6). The median Ct value of N genes was 23.85 (IQR, 18.70–28.70) (Figure 6). Of the 92 confirmed cases enrolled in the analysis of Ct value, the median Ct value of ORF1ab genes among cases aged 0–19 years, 20–59 years, and older than 59 years was 31.30 (IQR, 24.05–34.42), 23.90 (IQR, 20.15–28.25), and 25.80 (IQR, 21.25–27.30), respectively. The median Ct values of N genes among cases aged 0–19 years, 20–59 years, and older than 59 years were 31.62 (IQR, 22.22–34.30), 22.60 (IQR, 17.85–27.05), and 24.50 (IQR, 20.05–26.20), respectively. Both the median Ct values of ORF1ab genes and N genes among different age groups showed statistical significance (Figure 6). The median Ct values of the ORF1ab genes and the N genes among male cases (ORF1ab genes: median 25.90, IQR 22.38–31.47; N genes: median 24.85, IQR 20.90–30.92) were all higher than those among female cases (ORF1ab genes: median 23.65, IQR 19.95–28.95; N genes: median 22.50, IQR 17.87-27.55) but showed no statistical significance (Figure 6).

## Discussion

In this study, we described the epidemiological characteristics of SARS-CoV-2 Delta variant transmission in a well-traced local outbreak. The case with the earliest date of symptom onset in this outbreak was 13 July 2021, and the first case was identified and reported on 20 July 2021. Therefore, the hidden transmission may have occurred in this period.

We found that the estimated serial interval in this Delta outbreak was somewhat longer than those in Guangdong province (2.3 days) (14) and South Korea (3.26 days) (15). In both outbreaks caused by the Delta variant, the estimated serial intervals were shorter than those reported in the previous epidemic in Wuhan (7.5 days) (16) and Guangzhou (5.8 days) (17). A 4.2% negative serial interval proportion signifying presymptomatic transmission to some degree was also observed, which was considerably lower than that reported in a recent Delta outbreak in Guangdong province (4.2% vs. 21.6%) (14) but comparable to that reported by He et al. (4.2% vs. 7.6%) in the earlier COVID-19 epidemic stages, 2020 (17). When we considered the impact of gender and age on the serial interval, we did not observe significant associations between the serial interval with gender and age distribution. While the small sample size of infector–infectee pairs in some specific subgroups may contribute to the challenge to interpret the findings of our subgroup analysis. Further studies with larger sample sizes are needed to verify these findings.

The overall household SAR was 27.35% in this Delta variant outbreak. It was similar to the household SAR (22.0%) recently reported a Delta variant outbreak in Guangdong province (18). A much higher SAR (63.4%) was reported in South Korea without depicting the method in detail (15). The household SARs in both Delta variant outbreaks were higher than those reported in the early stages of the COVID-19 epidemic, 2020, which was estimated to be 17.1% when household contacts were defined on residential addresses (19). We did not observe a significant difference in the household SAR between household contacts exposed to male and female index cases. Our results were similar to the findings from a study in Guangzhou, China, which reported no significant differences in both household and non-household SARs of the original SARS-CoV-2 strain between different genders of the index cases (19). Oon Tek Ng et al. (20) reported that among the Delta variant-exposed household contacts, the male index cases (adjusted odds ratio (aOR), 0.86; 95% CI, 0.49–1.49) had no significant association with the reduced risk of SARS-CoV-2 acquisition. Meanwhile, another study (21) by Oon Tek Ng et al. showed that male index cases had a significant association with an increased risk of SARS-CoV-2 acquisition (aOR, 1.23; 95% CI, 1.04–1.46) among the Delta variant-exposed household contacts. More studies are needed regarding the association between the gender of the index cases and household SAR. A study from Singapore found that older index cases had more secondary infections than younger cases (aOR, 1.20 per decade; 95% CI, 1.03–1.39) among 753 household contacts linked to Delta variant index cases (20). However, there was no significant association determined for age grouped per decade of the index cases with the increased risk of SARS-CoV-2 acquisition of household contacts in our observed data.

Viral load was estimated in this outbreak because the high viral load was related to increased transmissibility. The Ct value serves as a proxy of viral concentration, and a lower Ct value of specimens indicates a higher amount of viral load within an infected individual and vice versa (22–24). The median Ct value of ORF1ab genes was similar to that reported in the Delta outbreak in Guangzhou (mean, 24.00; IQR, 19.00–29.00) (7). The Ct values were lower than those observed in the previously identified cases before the Delta variant emerged (data not shown), although caution should be exercised to compare the Ct values across studies due to multiple factors such as the time of sample collection. A report from India also demonstrated a higher proportion of positive samples with low Ct values during the second wave in 2021 caused by the Delta variant compared with that in the first wave in 2020 (25). Adult cases (20–59 years) showed lower Ct values of both ORF1ab genes and N genes than the cases among the youngest age group (0–19 years) and the oldest age group (≥60 years). A study based on the Delta variant outbreak in Guangdong showed that a complete primary series vaccination with two doses of the inactivated vaccines could effectively reduce the viral load in cases infected with the Delta variant and further lead to lower transmissibility (18).

The R_0_ estimated in this outbreak was similar to that reported in the Delta outbreak in Guangzhou (3.2, 95% CI, 2.0–4.8) (14). In a recent meta-review, the mean R_0_ value for the Delta variant was 5.08, ranging from 3.2 to 8.0 (10). The early estimate of R_0_ in China was 2.2 (95% CI, 1.4–3.9) for the previous variant from the early phase of the COVID-19 epidemic (16). Judging the threshold (*H* = *1–1/R*_0_), which was required to reach the estimated herd immunity, the coverage of vaccination should be promoted (10). The R_t_ value showed a continued declining trend with no apparent fluctuation observed, indicating that the comprehensive non-pharmaceutical interventions (NPIs) have been implemented effectively as an important public health tool in response to this COVID-19 outbreak in Nanjing. It should be noted that the estimates for epidemiological parameters like R_0_ and R_t_ in this outbreak did not take the potential influence factors, such as vaccination coverage and demographics, into account in the mathematic models. We nevertheless presented the parameters in a COVID-19 outbreak with the implementation of interventions in a real situation, which is helpful for understanding the real-world transmission of the Delta variant under the routine prevention and control measures during the COVID-19 epidemic.

Vaccination can prevent transmission both by blocking cases and by decreasing the risk of secondary cases from a vaccinated index case (26). A study (18) enrolled 5,153 close contacts of 73 index cases from a Delta variant outbreak and found that unvaccinated index cases (aOR, 2.84; 95% CI, 1.19–8.45) were more likely to transmit the infection to their contacts than those who are fully vaccinated (18). During the pre-Delta era, Harris et al. observed that the likelihood of household transmission was lowered by ~40–50% in vaccinated index cases (27).

The estimates of SARs varied depending on multiple factors, including the vaccination status. A study found that unvaccinated individuals exposed to the Delta variant showed an overall increased SAR of 7.8% (95% CI, 5.6–10%), a household SAR of 9.9% (95% CI, 5.8−14%), and an occupational SAR of 9.5% (95% CI, 0.8–18.1%) (28) compared with vaccinated individuals. Meanwhile, this study (28) found that there was no significant difference between the vaccination status for the estimation of the mean serial interval. The mean serial interval among unvaccinated and vaccinated index case-patients was 5.4 (SD: 3.1) and 5.3 (SD: 3.1), respectively (28). A meta-analysis presented SARs' estimates for different VOC variants, including the Delta variant, demonstrating that vaccination status affected SARs, which were higher from unvaccinated contacts than from partially or fully vaccinated contacts (29).

In addition, studies also found that the vaccination status of contacts exposed to an individual with SARS-CoV-2 infection was also associated with the risk of COVID-19 acquisition of contacts (20, 28). Among contacts exposed to the Delta variant, there is a lower risk of being infected by SARS-CoV-2 for fully vaccinated compared to unvaccinated contact and a lower risk of being infected by symptomatic disease in both fully and partially vaccinated contacts (20).

When estimating the value of R_0_, social contact behaviors and seroprevalence are usually taken into account if the corresponding data are available and reliable (30). A study (31) showed that close contacts with unmasked exposure (aOR, 4.9; 95% CI, 1.4–31.1) were associated with an increased risk of SARS-CoV-2 acquisition than those with only masked exposure. The protective role of masking has been shown in multiple settings (26). Since the COVID-19 epidemic broke out, mask-wearing has been an effective strategy to curb the spread of infections, and face masks are commonly used by the public (32) in China. During this outbreak, citizens are strongly advised to wear masks in public places, which as one of the NPIs may shrink the reproduction number, as reported *by* Stutt Rojh et al. (33).

This study explored the dynamic characteristics of an outbreak associated with the SARS-CoV-2 Delta (B.1.617.2) variant. Our findings provide a better understanding of the transmission of the Delta variant. Thus, continuous NPIs are still needed. As shown by the rapid reduction in R_t_ values in the study, multiple NPIs adopted in this outbreak showed very positive effectiveness in battling against the even highly infectious SARS-CoV-2 variant. Household SAR in this outbreak of 27.35% suggested that the Delta variant was more transmissible in households than the previously identified SARS-CoV-2 strain. The high household SAR should be taken into account when deciding on whether a close contact is quarantined at home or not in some special circumstances. In addition, the low Ct value of an emergent variant may serve as a marker for its high viral load, predicting the future spread of infection. However, the study was subject to some limitations. The sample size of the infector–infectee pairs used in our study was small and limited the ability to perform subgroup analysis, as we only included the infector–infectee pairs with epidemiologically defined transmission chains. In the circumstance where multiple potential infection sources existed, transmission chains could be inferred based on genetic evidence using next-generation sequencing (NGS) approaches, which are not included in this study. In addition, as an observational study, there may have been unmeasured variables that could affect parameter estimates. For example, close contacts with underlying diseases may be more likely to contract the disease, which may affect the estimates of household SAR. Future studies should collect more data in detail and explore more complex dynamic models for quantitative analysis of the epidemiological parameter estimates with the potential influence factors.

## Conclusions

Variants of SARS-CoV-2 continue to emerge. Understanding the epidemiological parameters that indicate the transmission dynamics of COVID-19 is essential for public health intervention, which could provide a reference for epidemiological analysis and the shape of control measures in outbreaks on a comparable or smaller scale caused by the Delta variant occurring in other cities in mainland China (34). Our findings offer new evidence to support the increased transmissibility of the SARS-CoV-2 Delta (B.1.617.2) variant. Although vaccination roll-out continues, NPIs will continue to play an important role, as new variants continue to rise in frequency.

## Data availability statement

The datasets presented in this article are not readily available because the license restrictions and the permission of Nanjing Municipal Center for Disease Control and Prevention. Requests to access the datasets should be directed to the corresponding authors or the author JW with the email wjunjun0316@163.com.

## Ethics statement

The epidemiological investigation was undertaken according to the Protocol for Prevention and Control of COVID-19. Individual-identifying information was not retained in analytic data sets.

## Author contributions

JD and NZ conceived and designed the study, coordinated data collection, reviewed, and revised the manuscript. JW, TM, SD, and KX conducted the literature search, carried out data analysis and interpretation, and drafted the manuscript. JW, TM, SD, and HF designed the data collection and performed quality control of data collection. MH and XD conducted laboratory experiments. MZ, ZZ, QD, ST, HW, XiC, ZF, HY, RW, CX, YX, LL, XuC, CL, WW, SYe, and SYa conducted the field investigations and collected data. All authors approved the version to be submitted.

## Funding

This work were supported by the Nanjing Key Medical Subject (Infectious Disease Prevention and Control) and the Project of Nanjing Health Science and Technology Development (YKK22190).

## Conflict of interest

The authors declare that the research was conducted in the absence of any commercial or financial relationships that could be construed as a potential conflict of interest.

## Publisher's note

All claims expressed in this article are solely those of the authors and do not necessarily represent those of their affiliated organizations, or those of the publisher, the editors and the reviewers. Any product that may be evaluated in this article, or claim that may be made by its manufacturer, is not guaranteed or endorsed by the publisher.
